# Unlocking epigenetic breeding potential in tomato and potato

**DOI:** 10.1007/s42994-024-00184-2

**Published:** 2024-10-23

**Authors:** Pingxian Zhang, Yuehui He, Sanwen Huang

**Affiliations:** 1 National Key Laboratory of Tropical Crop Breeding, Shenzhen Branch, Guangdong Laboratory of Lingnan Modern Agriculture, Genome Analysis Laboratory of the Ministry of Agriculture and Rural Affairs, Agricultural Genomics Institute at Shenzhen, Chinese Academy of Agricultural Sciences, Shenzhen, 518120 China; 2 Peking-Tsinghua Center for Life Sciences & State Key Laboratory of Wheat Improvement, School of Advanced Agricultural Sciences, Peking University, Beijing, 100871 China; 3 Peking University Institute of Advanced Agricultural Sciences, Shandong Laboratory of Advanced Agricultural Sciences in Weifang, Weifang, 261325 China; 4 National Key Laboratory of Tropical Crop Breeding, Chinese Academy of Tropical Agricultural Sciences, Haikou, 571101 China

**Keywords:** Epigenetic breeding potential, *Solanum* ENCODE, Synthetic epigenetics, AI-based epigenetic predictor, Epigenetic editing, Environmental adaptation

## Abstract

Tomato (*Solanum lycopersicum*) and potato (*Solanum tuberosum*), two integral crops within the nightshade family, are crucial sources of nutrients and serve as staple foods worldwide. Molecular genetic studies have significantly advanced our understanding of their domestication, evolution, and the establishment of key agronomic traits. Recent studies have revealed that epigenetic modifications act as “molecular switches”, crucially regulating phenotypic variations essential for traits such as fruit ripening in tomatoes and tuberization in potatoes. This review summarizes the latest findings on the regulatory mechanisms of epigenetic modifications in these crops and discusses the integration of biotechnology and epigenomics to enhance breeding strategies. By highlighting the role of epigenetic control in augmenting crop yield and adaptation, we underscores its potential to address the challenges posed by a growing global population as well as changing climate.

## Introduction

Chromatin serves as a substrate for a complex array of chemical modifications to DNA and histone proteins, which play pivotal roles in regulating genome structure and gene transcription. These dynamic modifications not only orchestrate the recruitment of effector protein complexes but also act as primary drivers for various biological processes. Epigenetic studies elucidate how alternative gene activity states can persist despite identical DNA sequences in each cell from a eukaryotic organism, with significant influence on genetic outcomes (Goldberg et al. 2007). Common epigenetic modifications involve “writers”, “erasers”, and “readers,” responsible for installing, removing, and recognizing epigenetic marks, respectively. In eukaryotes, mounting evidence underscores the crucial role of various epigenetic marks in modulating biological pathways, including stem cell division, embryonic development, sex differentiation, aging, oncogenesis, tumorigenesis, and environmental adaptation (Allis and Jenuwein 2016).

In plants, epigenetic regulation plays a pivotal role in interpreting developmental cues and facilitating adaptation to environmental changes (Andrés and Coupland 2012; He and Li 2018; Hou et al. 2022). For instance, in the model flowering plant *Arabidopsis thaliana*, histone modifications such as histone 3 lysine-4 tri-methylation (H3K4me3) and H3K27me3 are pivotal in regulating the key flowering-time genes *FLOWERING LOCUS C* (*FLC*) and *FLOWERING LOCUS T* (*FT*), thus controlling of the developmental transition to flowering in response to prolonged cold exposure in temperate winter seasons or photoperiodic signals (Gao and He 2024). This emphasizes the potential application of epigenetic-regulatory mechanisms in crop improvements.

In the Solanaceae family, particularly tomato (*Solanum lycopersicum*) and potato (*Solanum tuberosum*), epigenetic regulation has emerged as a crucial factor in modulating agronomic traits. Tomato, the world’s largest vegetable crop, and potato, the third staple food globally, both are under epigenetic regulation in vital processes, encompassing fleshy fruit ripening, floral development, and tuberization (Giovannoni et al. 2017; Tang et al. 2020; Kondhare et al. 2021). This review summarizes the latest advancements in epigenetic regulation in tomato and potato. It highlights cutting-edge research in the modulation of developmental processes, adaptation to environmental changes, and breeding strategies. This paper underscores the significance of these studies and their immense potential to further our understanding of epigenetics, as well as its breeding applications in tomato and potato.

## Histone modifications

**Fig. 1:**
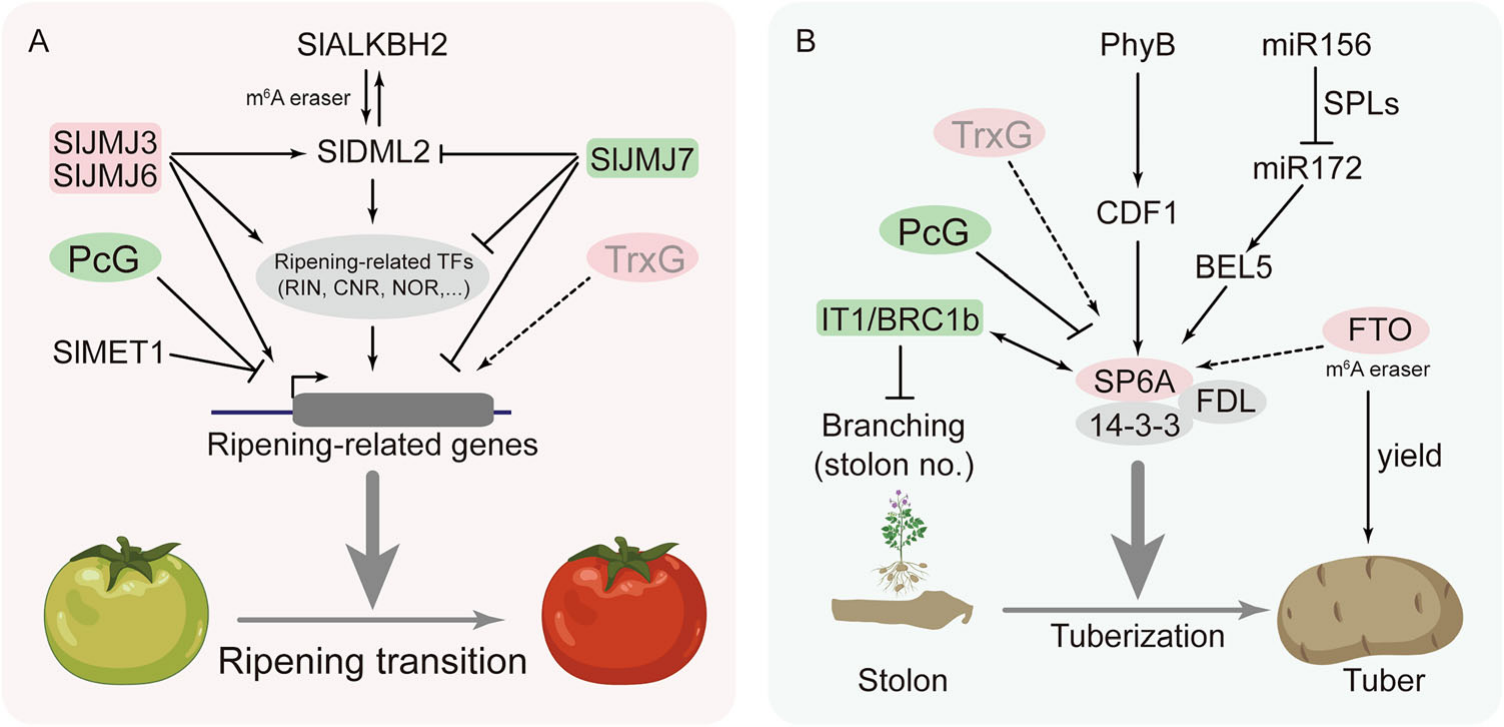
Epigenetic regulatory mechanisms of tomato fruit ripening (**A**) and potato tuberization (**B**). Recent studies have shown that in tomato, histone regulators such as the PcG complex, JMJs, DNA 5mC factors like MET1 and DML2, as well as RNA m^6^A modification mediated by ALKBH2, play a crucial role in fruit ripening. Conversely, in potato, only PcG factors and microRNAs (miRNAs) have been identified as pivotal players in orchestrating the stolon-to-tuber transition. The figure elements were sourced and adapted from BioRender.com

Histone post-translational modifications (PTMs) organize chromatin and contribute to gene regulation. These PTMs are installed or removed by histone-modifying enzymes, such as histone methyltransferases (HMTs), histone demethylases, and their expression fluctuates during developmental phases, thereby influencing chromatin structure and stability (Liu et al. 2010). Histone PTMs play a pivotal role in regulating gene expression across diverse developmental stages, significantly influencing critical agronomic traits such as fruit ripening and tuberization (Fig. 1).

### Fruit ripening

As a climacteric fruit, tomato ripening is intricately orchestrated by hormones, transcription factors (TFs), and epigenetic modifications, and thus to regulate gene expression with the ripening process (Brumos 2021). In tomato, a distinct type of HMTs to catalyze H3K27me3, known as the Polycomb Repressive Complex 2 (PRC2), has been identified to regulate fruit development and ripening (Kim et al. 2023). For example, the genes encoding ENHANCER OF ZESTE (E(z)) proteins, homologs of the *Arabidopsis* CURLY LEAF (CLF) H3K27 methyltransferase, are preferentially expressed during the early stages of fruit development, underscoring the role of histone methylation in cell division and early developmental processes (Kit et al. 2010; Boureau et al. 2016). Overexpression of *MSI1*, another PRC2 component known as *MULTICOPY SUPPRESSOR OF IRA1*, leads to the suppression of ripening-related gene expression, thereby impeding the fruit ripening process in tomato (Liu et al. 2016). Similarly, the *VERNALIZATION INSENSITIVE 3-LIKE* gene *CRAWLING ELEPHANT* (*CREL*) controls fruit maturation and differentiation in tomato through Polycomb silencing (Almutairi and Sadder 2014; Shwartz et al. 2022).

Furthermore, the H3K27me3 reader protein LIKE HETEROCHROMATIN PROTEIN 1 (LHP1), a member of the Polycomb Repressive Complex 1 (PRC1), plays a regulatory role in fruit development and ripening in tomato. Tomato LHP1 exists in two variants, SlLHP1a and SlLHP1b. SlLHP1b binds to the H3K27me3 mark in the regions of ripening-associated genes involved in ethylene and carotenoid biosynthesis, as well as *RIPENING INHIBITOR* (*RIN*) targeted genes, effectively suppressing fruit ripening. Additionally, SlLHP1b interacts with SlMSI1, hinting a potential role of these two Polycomb complexes in regulating tomato fruit ripening (Liang et al. 2020). Although other PRC1 RING-finger proteins, BMI1A/B/C, have been shown to play a role in axillary meristem formation and leaf development in tomatoes (López et al. 2021), the question of whether they are essential for fruit ripening remains unanswered. Similarly, the Trithorax group (TrxG) of HMTs, known for catalyzing H3K4me3, a pivotal epigenetic mark in active gene transcription (Pu and Sung 2015), has yet to be thoroughly examined for its individual contributions to tomato fruit ripening (Fig. 1A).

Histone methylation is dynamically regulated by HMTs and histone demethylases such as Jumonji C domain-containing demethylases (JMJs). The histone demethylase SlJMJ6 has been demonstrated to foster tomato fruit ripening by facilitating the demethylation of the inhibitory histone mark H3K27me3 (Li et al. 2020). Overexpression of *SlJMJ6* sparked the activation of ripening-related genes, subsequently hastening the progression of tomato fruit ripening (Li et al. 2020). Similarly, the overexpression of the additional H3K27me3 demethylase *SlJMJ3* expedited the fruit ripening process in tomatoes, whereas the loss-of-function mutation in *SlJMJ3* led to a delayed ripening process (Li et al. 2024b). A third H3K27 demethylase *SlJMJ4* has been shown to promote dark- and abscisic acid-induced leaf senescence in tomato, but its role in fruit ripening remains unclear (Ding et al. 2022b). Furthermore, apart from H3K27 demethylases, the H3K4me3 demethylase SlJMJ7 also exerts regulatory functions in tomato fruit ripening. By demethylating the active histone mark H3K4me3, SlJMJ7 triggers the downregulation of ripening-related genes associated with ethylene biosynthesis, thus resulting in a delayed ripening process of tomato fruits (Ding et al. 2022a). These examples indicate that in the regulation of tomato fruit ripening, the interplay between HMTs and JMJs can either activate or repress gene expression, contingent upon the specific type of histone methylation that is being catalyzed or erased.

Furthermore, several master transcription factors, such as COLOURLESS NON-RIPENING (CNR), NON-RIPENING (NOR), and RIN, play a pivotal role in positively regulating tomato fruit ripening (Brumos 2021). Recent studies have uncovered that during fruit maturation, the deposition of epigenetic marks in these loci augmented, whereas the repressive H3K27me3 marks in particular, has played a conserved role in restricting ripening genes and their counterparts in tomato fruits (Lü et al. 2018; Gao et al. 2019). In addition, the transcription factor Nuclear Factor Y (NF-Y) has been revealed as a crucial regulator of tomato fruit ripening, acting in coordination with Polycomb repression. Reduced expression of *NF-YB* genes results in elevated levels of H3K27me3 at the *CHS1* (*CHALCONE SYNTHASE 1*) locus, and thus decreased expression of *CHS1* (Wang et al. 2021a). This inhibition of *CHS1* expression impedes flavonoid accumulation, resulting in fruits with pink color and colorless peels (Wang et al. 2021a).

### Tuberization

Potato undergoes a unique process known as tuberization, where specialized below ground branches, called stolons, differentiate through radial expansion of their terminal ends. The formation of underground tubers in potatoes has become a model system for understanding the control of storage organ formation. In addition to phytohormone and sugar signaling, recent studies have revealed two other key factors that contribute to the transition from stolon to tuber: photoperiod and epigenetic modifiers (Kondhare et al. 2021). The main photoperiodic pathway implicated in the stolon-to-tuber transition encompasses PHYTOCHROME B (PHYB), CYCLING DOF FACTOR 1 (CDF1), and SELF-PRUNING 6A (SP6A) (Zierer et al. 2021). In addition, this transition is intricately regulated by several crucial transcriptional factors, including Identity of Tuber1 (IT1) /BRANCHED1b (BRC1b) (Nicolas et al. 2022; Tang et al. 2022), and the mobile BEL1-like protein BEL5 (Banerjee et al. 2006).

In recent years, epigenetic regulators such as PcG factors and microRNAs (miRNAs) have been shown to play pivotal roles in orchestrating this transition (Fig. 1B). For example, the PRC2 component StE(z)2 functions to repress the expression of an important tuberization inducer, *StSP6A*, in leaves through the deposition of H3K27me3, thereby inhibiting the belowground tuber formation in long days. However, under tuber-inductive short-day conditions, the reduction of H3K27me3 at the *StSP6A* locus leads to the induction of its expression and subsequent development of belowground tubers (Kumar et al. 2021). Another study has showed that the PRC2 subunit StMSI1 and the PRC1 protein StBMI1 (Kumar et al. 2020), function upstream of miR156, to control the formation of aerial tubers in potato (Bhogale et al. 2014). Overexpression of *StMSI1* or knockdown of *StBMI1* expression resulted in increased miR156 levels and the formation of aerial stolons and tubers under short-day photoperiods (Kumar et al. 2020).

### Responses to biotic and abiotic stresses

With climate change posing significant challenges to global food security, the ability of plants to respond effectively to biotic and abiotic stresses has become a pressing concern. Epigenetic modifications offer the potential to improve stress and pathogen tolerance without compromising growth, by modulating epigenetic chromatin marks. For example, in tomato, the reductions of H3K4me3 and H3K36me3 at regulatory loci contribute to biotic and abiotic stress responses, and functional losses of two histone methyltransferases, SET Domain Group 33 (SDG33) and SDG34, have been shown to be implicated in stress tolerance (Bvindi et al. 2022). Similarly, in potato tubers, a genome-wide enrichment of H3K27 acetylation (H3K27ac) has been found to be associated with enhanced resistance to cold-induced sweetening (Guo et al. 2023). These findings highlight the potential of integrating various histone modifications for adaptation to stresses in tomatoes and potatoes.

## DNA methylation

Cytosine DNA methylation (5-methylcytosine, 5mC) is a crucial and conserved epigenetic mark involved in various biological processes, including gene and transposon silencing, maintenance of genome stability, developmental regulation, and responses to environmental stresses (Law and Jacobsen 2010). Unlike mammals, where DNA methylation that occurs almost exclusively in the symmetric CG context, methylation in plants has evolved in three sequence contexts on cytosine bases: the symmetric CG and CHG contexts (H for A, T, or C), and the asymmetric CHH context (Henderson and Jacobsen 2007). In plants, the levels of 5mC, which reflect the dynamic modulation of establishment, maintenance, and removal activities, are crucial for understanding epigenetic regulation across these three cytosine sequence contexts. In *Arabidopsis*, de novo methylation is catalyzed by DOMAINS REARRANGED METHYLTRANSFERASE 2 (DRM2) and DRM1 through the RNA-directed DNA methylation (RdDM) pathway (Matzke and Mosher 2014). In contrast, the maintenance of DNA methylation depends on DNA METHYLTRANSFERASE 1 (MET1), CHROMOMETHYLASE 3 (CMT3), CMT2, and the RdDM pathway (Zhang et al. 2018). DNA methylation can be actively removed by specific enzymes or passively lost due to the failure to maintain methylation after DNA replication. In plants, the REPRESSOR OF SILENCING 1 (ROS1) family proteins, which are 5mC DNA glycosylases/lyases (demethylases), are considered to be key players in active DNA demethylation (Zhang et al. 2018).

### Dynamic DNA methylation

The dynamic DNA methylation pattern is determined by the antagonistic activities of DNA methylation and active demethylation. Changes in the balance between these processes alter the DNA methylation status of the genome.

During the ripening of tomato fruits, a notable decrease of approximately 20–30% in global DNA methylation levels has been observed across the entire genome (Giovannoni et al. 2017). This demethylation trend is particularly evident in the pericarp tissues, underscoring its potential significance in fruit maturation. This transformative demethylation process exerts an impact on specific DNA sequences and chromatin structural contexts that regulate the expression of ripening-related genes. Remarkably, the general pattern of demethylation occurs in harmony with the timing of the mature green tomato fruit acquiring the capability to ripen in response to exogenous ethylene, signifying a precise orchestration between epigenetic changes and the critical transition towards fruit ripening (Zhong et al. 2013). While epigenetic mechanisms by DNA methylation undoubtedly contribute to the regulation of potato tuberization, the specific role of dynamic DNA methylation patterns in tuber initiation and growth has not been well defined.

In tomatoes, protein sequence homology with *Arabidopsis* DNA demethylases suggests the presence of four putative DNA demethylases: SlDML1 to SlDML4. Among which SlDML1 and SlDML2 are most closely related to the *Arabidopsis* ROS1 (Liu et al. 2015). Knockdown of *SlDML2* expression results in significant decreases in various gene expression and alters the methylation status of the fruit (Liu et al. 2015). Loss-of-function mutations in *SlDML2* lead to hypermethylation across thousands of genes, including those involved in ethylene production, pigment synthesis, and cell wall hydrolysis during ripening (Lang et al. 2017). Furthermore, *sldml2* mutants, in addition to developmental defects, have been found to be more susceptible to the necrotrophic fungal pathogen *Botrytis cinerea* (Zhou et al. 2023)*.* Intriguingly, a very recent study has revealed that downregulating the potato equivalent, *StDML2*, can modulate herbivore defense mechanisms by influencing Jasmonate signaling and the production of defense compounds (Zhang et al. 2024a). These collective results underscore the pivotal role of active DNA demethylation, particularly mediated by DML2 in tomatoes for fruit ripening and, and for defense against diseases and insect pests in both tomato and potato.

Conversely, a recent study has shown that the DNA methylase SlMET1 is strongly associated with developmental processes through its regulation of DNA methylation in tomato (Yang et al. 2019). A frame-shift mutation of *SlMET1* caused severe developmental defects, such as curly leaves, defective inflorescence, and parthenocarpy. In tomato, *slmet1* mutations resulted in CG hypomethylation and CHH hypermethylation on a genome-wide scale, disturbing transcription regulation of downstream targets. These findings underscore the complexity of how DNA (de)methylation contributes to ripening and development-associated alterations in DNA methylation patterns.

### Locus-specific DNA methylation

Diverse biological processes have been regulated by locus-specific DNA methylation in tomato and potato. In tomato fruits, for example, DNA demethylation at promoter regions of ripening-related genes facilitates the binding of TFs, thereby activating pathways critical for ethylene production, pigment biosynthesis, and cell wall modification (Shinozaki et al. 2018). A milestone study has revealed that a natural epigenetic mutation known as *Cnr*, inhibits normal fruit ripening, resulting in fruits with a colorless and mealy pericarp due to DNA hypermethylation in the *CNR* promoter region (Zhong et al. 2013). The *Cnr* phenotype is caused by the hypermethylation of the 286 bp region located 2.4 kb upstream of a SQUAMOSA promoter-binding protein-like (SBP box/SPL) transcription factor, leading to *CNR* repression (Manning et al. 2006).

Furthermore, naturally occurring variations in the vitamin E content in tomato fruits has been found to be associated with changes in DNA methylation at a promoter region of the 2-methyl-6-phytylquinol methyltransferase gene [*VTE3* *(1)*] (Quadrana et al. 2014). Similarly, it has been shown that CG hypermethylation of the *bHLH39* promoter is associated with its expression and Fe deficiency responses in tomato roots (Zhu et al. 2023).

In terms of potato, Drozda et al. (2022) have underscored the central role of the RdDM pathway in fine-tuning the expression of the *R3a* gene, a key player in conferring resistance against late blight disease. Collectively, these findings highlight the profound impact of epigenetic regulation by DNA methylation, particularly at specific regulatory loci, on diverse biological functions and resilience in tomato and potato.

### Response to environmental factors

Studies have uncovered a robust link between DNA methylation and responses to environmental factors in tomato and potato. This is particularly notable in the chilling-induced flavor loss in tomato, which is associated with altered volatile synthesis and transient changes in DNA methylation (Zhang et al. 2016). Similarly, under phosphate starvation conditions in tomato, the alterations of DNA methylation have been shown to correlate with alterations in gene expression and alternative splicing (Tian et al. 2021). Analogously, in potatoes, tuberization genes have been shown to be regulated by DNA methylation in response to high temperature, and light exposure (Ai et al. 2021; Dutta et al. 2022; Xiong et al. 2022). These findings collectively suggest that epigenetic mechanisms via DNA methylation hold promise for environmental adaptability.

### A transposon defense system by DNA methylation

In plant lineages, bursts of transposable elements (TEs) are common occurrences. DNA methylation is essential for silencing these TEs through a defense system of genomic immunity in plants (Kim and Zilberman 2014). Initially, the silencing of TEs is established by RdDM, and the maintenance of TE silencing relies on the coordination of RdDM and RNA-independent DNA methyltransferases, such as MET1, CMT2, and CMT3. A recent study has analyzed the DNA methylome landscape across plant lineages (including tomato and potato) and revealed that recently transposed TEs were more heavily associated with DNA methylation compared to ancient ones (Wang et al. 2019). Specifically, in tomato, preferential CHG methylation of younger long terminal repeats (LTRs) transposons are mediated by CMTs (Wang and Baulcombe 2020). These CMTs silence LTRs in distal chromatin regions that are younger than those controlled by RdDM. Additionally, a genome-wide redistribution of CHH methylation was also observed in tomato *ddm1* mutants (Corem et al. 2018). Another study reveals that *Rider* retrotransposon activity in tomato is triggered by drought stress and controlled by epigenetic mechanisms, contributing to phenotypic variation and suggesting *Rider* as a potential source of genetic and epigenetic diversity (Benoit et al. 2019). The evolution of transposed TEs may also be driven by natural environmental factors via DNA methylation regulation.

## RNA modifications

RNA modifications, particularly *N6*-methyladenosine (m^6^A) and 5-methylcytosine (m^5^C), play essential roles in regulating mRNA stability and thus influence various developmental processes and stress responses in plants. Recent research in tomatoes has shown dynamic changes in m^6^A levels during fruit ripening, mirroring patterns observed in DNA methylation (Zhou et al. 2019). In the *Cnr* mutant, which exhibits increased DNA methylation, an increase in m^6^A was observed in over 1100 transcripts, indicating a global upregulation of this modification (Zhou et al. 2019). This increase in m^6^A is correlated with decreased expression of the RNA demethylase gene *SlALKBH2*, which also affects the stability of the DNA demethylase *SlDML2* mRNA, crucial for tomato ripening (Zhou et al. 2019). Another study revealed that several m^5^C-modified mRNA targets are involved in tomato fruit ripening, which is associated with differentially 5mC-methylated regions and DEGs related to ethylene biosynthesis and signal transduction (Guo et al. 2022a). In potato, transgenic expression of the human RNA demethylase FTO (Fat Mass and Obesity-Associated Protein) caused ~ 50% increases in yield and biomass, demonstrating the potential of RNA modification for crop improvement (Yu et al. 2021). More recently, an m^6^A reader protein in tomato, SlYTH2, has been revealed to regulate fruit ripening and quality by promoting RNA–protein condensate formation, accelerating translation of aroma-related genes, and thus to enhance consumer preferences (Bian et al. 2024). These studies suggest that modulating RNA modifications is a promising strategy for the enhancement of growth and yield in tomato and potato. Further research into the mechanisms by which RNA modifications control gene expression and plant development will be essential for developing targeted interventions to improve crop performance.

## Strategies unlocking epigenetic breeding potential

### *Solanum* ENCODE project

A myriad of epigenetic modifications on DNA and histone protein tails orchestrate gene expression, exerting either repressive or activating effects while shaping chromatin accessibility—a balance between condensed and open configurations. Specifically, H3K4me3 typically correlates with gene activation in more accessible chromatin regions, whereas DNA 5mC, and H3K27me3 are frequently associated with chromatin condensation and gene repression. Beyond these classical modifications, recent advances have illuminated the pivotal role of RNA m^6^A as an additional layer of regulation, influencing both gene expression and translational efficiency (Fu et al. 2014; Murakami and Jaffrey 2022).

Within the nucleus, these epigenetic marks are intricately organized in a hierarchical three-dimensional (3D) structure, facilitating topological interactions among proximal and distal DNA segments, as well as between DNA and RNA, to orchestrate transcriptional regulation. This intricate landscape poses a fundamental query in plant biology: How do these complex epigenetic marks, in concert with transcription factors, precisely control the expression levels of target genes? At the heart of this inquiry lie *cis*-regulatory DNA elements (CREs), which serve as binding sites for transcription factors (TFs).

CREs are further organized into diverse cis-regulatory modules (CRMs), a strategic assembly comprising core promoters, enhancers, and silencers, strategically positioned along the chromosome (Schmitz et al. 2022). Each CRM performs a unique yet interconnected role in fine-tuning gene expression. Beyond the core promoters, which represent the minimal sequence region essential for initiating transcription and typically span 50–100 base pairs around the transcription start site (TSS), other CRMs exhibit variability in their chromosomal locations, occurring upstream, downstream, intergenic, or even within introns (Schmitz et al. 2022). As one example for illustrating this diversity, a study in potato has unveiled a 200 bp intronic enhancer, *VInv*In2En, that controls cold-induced expression of the vacuolar invertase gene (*VInv*) in response to cold stress (Zhu et al. 2024). Thus, the genome-wide identification and functional characterization of these CREs and CRMs are paramount to unraveling the complexities of plant gene regulation.

To unravel the complexities of regulatory mechanisms, the ENCyclopedia of DNA Elements (ENCODE) project, initially established for humans, has broadened its horizons to encompass numerous eukaryotes, inspiring plant biologists to propose analogous initiatives tailored for plants (Lane et al. 2014). A fruit-centric ENCODE project, for instance, conducted a comprehensive analysis of epigenetic modifications across fruit developmental stages, partially uncovering negative correlation of H3K27me3 with the expression of ripening-related genes across various fruit species, particularly tomatoes (Lü et al. 2018). Recently, a study in tomatoes has highlighted the pivotal role of HSFA1a, a crucial TF for heat stress resilience, in orchestrating chromatin's spatial reorganization and modulating dynamic transcriptional responses via the 3D remodeling of promoter–enhancer contacts (Huang et al. 2023). Similarly, DNase I hypersensitive sites (DHSs) reveal active CREs in potato, and high-resolution DHS mapping under normal and cold storage identifies cold-induced DHSs enriched in genic regions, enhancing chromatin accessibility of active genes, including those marked with the bivalent marks of H3K4me3 and H3K27me3 (Zeng et al. 2019). Another comprehensive investigation delved further into the intricate interplay between H3K9ac and the 3D chromatin organization in tomato, thereby enhancing our understanding of these complex epigenetic-regulatory mechanisms (An et al. 2024). Collectively, these findings underscore the intricate interplay between epigenetic marks, chromatin structure, and transcriptional regulators, opening new avenues for understanding and manipulating developmental processes and environmental responses in tomato.

**Fig. 2:**
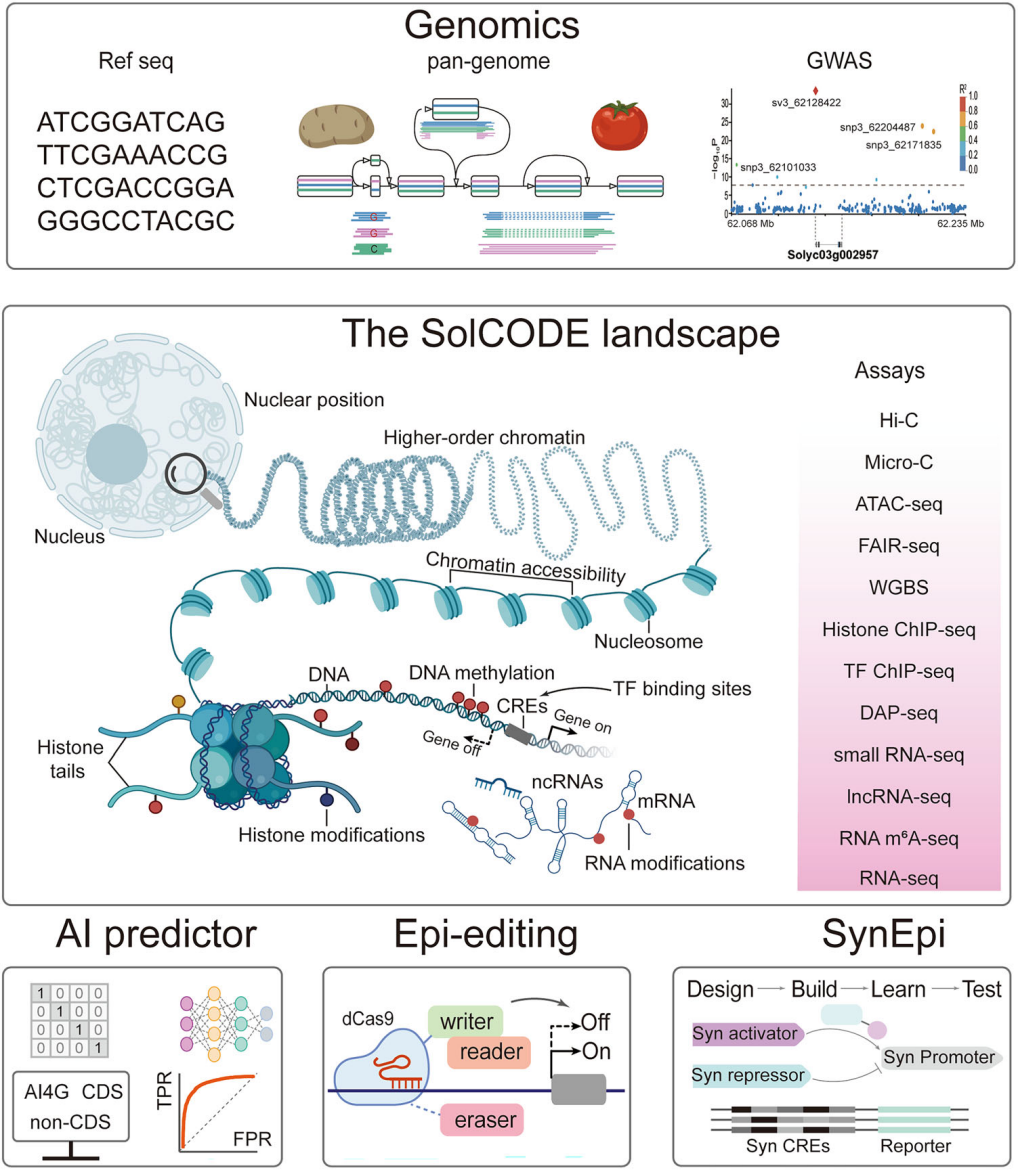
Tools for unlocking epigenetic breeding potential in tomato and potato. In these two nightshade crops, numerous genomic studies have already enhanced their breeding practices. Leveraging the existing robust infrastructure of genomic big data, we underscore the urgent need for a *Solanum* ENCODE (SolCODE) project. Such an initiative would be a treasure trove for the tomato and potato epigenetic research community, providing large-scale datasets encompassing cis-regulatory DNA elements (CREs), epigenetic marks, and the 3D organization of genomes. Along with downstream tools including AI predictors, epigenetic editing (Epi-editing), and synthetic epigenetics (Syn-Epi), these tools would pave the way for unraveling how the epigenomes of nightshade species encode agronomic traits. The figure elements were sourced and adapted from BioRender.com

In this context, we underscore the pressing need for a *Solanum* ENCODE (SolCODE) project (Fig. 2). Such an initiative would be a treasure trove for the tomato and potato epigenetic research community, offering large-scale datasets encompassing CREs, epigenetic marks, and the 3D organization of genomes. SolCODE would pave the way for unraveling how the genomes of nightshade species encode agronomic traits, leveraging our robust infrastructure of genomic big data (Zhu et al. 2018; Tang et al. 2022; Zhou et al. 2022; Usadel 2022; Wu et al. 2023). This endeavor would not only deepen our understanding of epigenomic functions but also facilitate the epigenetic-driven breeding of superior varieties, such as 'delicious tomatoes’ and hybrid diploid potatoes (Zhu et al. 2018; Comai 2023), thereby revolutionizing the agricultural landscape.

### AI-based epigenetic predictor

Deep learning, as a subset of artificial intelligence (AI), presents a promising avenue for advancing plant epigenomics. The capability of deep learning to extract novel features from complex data makes it well suited for plant biology and agricultural science (Wang et al. 2020; Chen et al. 2024).

Within the expansive field of plant biology and agricultural science, deep learning has emerged as a potent tool for predicting potential traits and epigenetic components after learning the current various epigenetic and epigenomic datasets. Specifically, deep learning is widely used to extract the epigenetic features based on epigenomic datasets and making an epigenetic prediction, such as histone modifications, RNA alterations, DNA 5mC patterns, and chromatin accessibility (Wang et al. 2021b; Guo et al. 2022b). Remarkably, in the case of tomato, deep learning has not only revolutionized the prediction of regulatory networks from CRE patterns and transcription factor interactions but also offered an approach to designing alleles tailored to optimize the gene expression level (Akagi et al. 2022). More significantly, used deep learning models to predict the gene expression profiles by training the non-coding sequences from various plant species (including tomato), with the accuracy over 80% CREs are able to make differences in gene expression levels (Peleke et al. 2024). Despite these advances, the inherent complexity of epigenetic-regulatory networks requires the efficient predictive tools for multi-epigenomic datasets to integrate and interpret the epigenetic phenomena within *Solanum* species.

### Editing epigenetic marks

The advent of clustered regularly interspaced short palindromic repeats (CRISPR)/CRISPR-associated proteins (Cas) genome-editing tools has revolutionized life sciences, introducing transformative capabilities across a broad spectrum of applications including nucleases, base editors, transposases/recombinases, and prime editors. Since its first application in plants in 2013, CRISPR/Cas has dramatically advanced genome editing in various crop species, incorporating valuable agronomic traits (Li et al. 2024a). Recent developments in catalytically inactivated Cas variants (dCas) have expanded the application of the CRISPR/Cas system for epigenetics studies and transcriptional and translational regulation without creating DNA double-strand breaks (DSBs), which promise substantial impacts on agriculture by enabling specific epigenomic modifications for crop improvement (Pan et al. 2021).

In mammals, a study leveraging CRISPRoff tool has demonstrated promising potential in regulating gene expression through manipulating DNA methylation and thus heritable gene silencing (Nuñez et al. 2021). Another fundamental epigenetic research development is the engineered CRISPR-Cas-based DNA adenine methylation (6mA) editing systems in human cells (Park et al. 2019). This tool implements synthetic factors of 6mA writers and readers, which can effectively recruit transcriptional regulators to regulate reporter loci.

Targeted gene silencing systems have been established through diverse epigenetic pathways in plant. A recent study has identified multiple regulatory proteins capable of gene silencing through distinct epigenetic mechanisms, demonstrating their potential in targeted gene manipulation via the artificial zinc finger (ZF) and dCas9-SunTag systems (Wang et al. 2023). Moreover, a DNA methylation-based gene silencing system has been applied in blocking host susceptibility (*S*) gene expression to enhance disease resistance in cassava (Veley et al. 2023). These technological breakthroughs in editing epigenetic marks not only promote fundamental epigenetic research, but also open doors for widespread applications across diverse biological studies such as crop improvement.

### Synthetic epigenetic regulation

As mentioned above with three breeding strategies, they offer robust platforms for the application of synthetic epigenetic regulation (SynEpi) systems, further broadening the scope of tomato and potato improvement. In eukaryotes, as synthetic biology (SynBio) becomes pivotal for gene regulation, a novel interdisciplinary field termed SynEpi has emerged, combining synthetic engineering principles with the principles and dynamic nature of epigenetic regulation. This approach aims to design and control spatial and temporal gene regulation in response to developmental factors and environmental inputs. One notable example is the above-described study by Park et al. (2019), in which a SynEpi regulatory system is constructed, using DNA 6 mA writer–reader modules.

In plants, researchers have developed complex gene editing-based systems that act as programmable digital logic gates for dynamic and reversible gene regulation, such as the CRISPR-Cas9 SunTag system (Papikian et al. 2019). These innovations highlight the potential of SynEpi regulation to engineer complex cellular functions and coordinate multiple cellular processes on a scale previously unachievable. Unlike traditional biotechnologies that primarily rely on recombinant DNA technology, plant SynEpi regulation integrates epigenetic-regulatory modules with synthetic systems in novel ways, constructing new pathways for plant biology and crop improvement. As this field continues to evolve, SynEpi will undoubtedly play a critical role in future studies and applications aimed at enhancing crop performance.

## Concluding remarks

Research into epigenetic modifications in tomato and potato stands as an evolving and rapidly progressing field, offering profound insights into the intricate mechanisms that govern growth, development, and adaptation to environmental cues in tomato and potato. Although significant advancements have been made in recent years, enriching our understanding of epigenetic regulation in these two economically important crops, the landscape of epigenetic research remains largely exploratory and fraught with untapped potential.

One of the key areas of focus in epigenetic research on tomato and potato is to understand the role of epigenetic factors in shaping critical agronomic traits. For instance, elucidating the epigenetic mechanisms underlying tomato fruit ripening and potato tuberization could lead to the development of varieties with improved quality and yield. By leveraging epigenetic regulators, breeders can potentially create more resilient and productive crops that are better adapted to changing environmental conditions, such as drought, heat stress, and disease outbreaks.

Moreover, epigenetic diversity within a species represents an untapped source of genetic variation that can be leveraged for crop improvement (Springer and Schmitz 2017). To fully harness the potential of epigenetic variations in tomato and potato, it is essential to develop and apply advanced methods for epigenome profiling and engineering (Fig. 2). These techniques will enable researchers to comprehensively map epigenetic marks across the genome and manipulate them to create desired traits. As the field of epigenetics continues to evolve, we can expect to see the development of new tools and strategies that will further expand our ability to harness epigenetic regulation for improvement of these crops.

It is worth noting that the endeavor of generating data in high starch-polysaccharide tissues and implementing gene editing across diverse tomato and potato varieties presents researchers with multifarious and intricate challenges. Researchers are harnessing technological advancements to navigate these complexities. For example, a recent study has established an advanced chromatin immunoprecipitation followed by sequencing (aChIP-seq) to efficiently isolate chromatins from starch-polysaccharide accumulated tissues (Zhang et al. 2024b). Other technical advances have refined the delivery systems for genetic transformation, thereby enhancing the effectiveness of gene editing across different varieties (Cao et al. 2022; Mei et al. 2024).

In summary, the ongoing research into epigenetic modifications in tomato and potato plants represents a promising frontier in agricultural science, with the potential to revolutionize our approach to crop breeding and management. By unlocking the epigenetic breeding potential of these crops, we stand poised to cultivate more sustainable, nutritious, and resilient varieties that can withstand the pressures of a changing world and contribute to global food security.

## Data Availability

Data sharing not applicable to this article as no datasets were generated or analysed during the current study.
